# From Acupuncture to Interaction between **δ**-Opioid Receptors and Na^**+**^ Channels: A Potential Pathway to Inhibit Epileptic Hyperexcitability

**DOI:** 10.1155/2013/216016

**Published:** 2013-04-03

**Authors:** Dongman Chao, Xueyong Shen, Ying Xia

**Affiliations:** ^1^The University of Texas Medical School at Houston, Houston, TX 77030, USA; ^2^Yale University School of Medicine, New Haven, CT 06520, USA; ^3^Shanghai Research Center for Acupuncture and Meridians, Shanghai 201203, China; ^4^Shanghai University of Traditional Chinese Medicine, Shanghai 201203, China

## Abstract

Epilepsy is one of the most common neurological disorders affecting about 1% of population. Although the precise mechanism of its pathophysiological changes in the brain is unknown, epilepsy has been recognized as a disorder of brain excitability characterized by recurrent unprovoked seizures that result from the abnormal, excessive, and synchronous activity of clusters of nerve cells in the brain. Currently available therapies, including medical, surgical, and other strategies, such as ketogenic diet and vagus nerve stimulation, are symptomatic with their own limitations and complications. Seeking new strategies to cure this serious disorder still poses a big challenge to the field of medicine. Our recent studies suggest that acupuncture may exert its antiepileptic effects by normalizing the disrupted neuronal and network excitability through several mechanisms, including lowering the overexcited neuronal activity, enhancing the inhibitory system, and attenuating the excitatory system in the brain via regulation of the interaction between **δ**-opioid receptors (DOR) and Na^+^ channels. This paper reviews the progress in this field and summarizes new knowledge based on our work and those of others.

## 1. Introduction

Epilepsy is one of the most common neurological disorders affecting about 1% of population. Currently available therapies, including medical treatment, surgical treatment, and other treatment strategies such as ketogenic diet and vagus nerve stimulation are symptomatic with their own limitations and complications [1]. Understanding of its cellular and molecular mechanisms and seeking new strategies to cure this disorder still poses a big challenge.

Epilepsy has been recognized as a disorder of brain excitability characterized by recurrent unprovoked seizures that result from the abnormal, excessive, and synchronous activity of clusters of nerve cells in the brain. About 40% of epilepsies are mainly caused by genetic factors, while the other 60% are acquired/etiological epilepsies. Irrespective of the inherited or acquired type, the changes in neuronal excitability that underlie the pathogenesis of epilepsy are a complex process that induces abnormal activity not only in individual neurons, but also in a population of hyperexcitable neurons in highly synchronous activities that are propagated through normal or pathological pathways.

Epileptic hyperexcitability results from multiple factors such as innate ability of neurons in the cortex and hippocampus, alterations in the membrane properties, imbalance between excitatory and inhibitory transmission, alterations in existing synaptic contacts/circuits and/or establishment of new synaptic contacts/circuits, and the inability of glial cells to maintain glutamate and K^+^ homeostasis [1]. Among these factors, the most important determinant is the intrinsic electrogenic property of each neuron that depends on the function of ion channels like Na^+^, K^+^, and Ca^2+^ channels in the membrane [2]. In particular, Na^+^ channels are responsible for the initiation and propagation of action potential, and are critical determinants of intrinsic neuronal excitability.

Acupuncture is one of the oriental medical therapeutic techniques that involves insertion of fine needles into specific body areas (acupoints or meridian points) and swift manual manipulation (e.g., rotating, lifting, thrusting, retaining, etc.) that results in the manifestation of the *Qi* or *De Qi *(acquisition of energy) phenomenon that refers to a mixed sensation of soreness, numbness, swelling, sinking, and heat that appears in the acupoints. *De Qi* is an important component of the therapeutic effect and may be necessary for clinical efficacy of acupuncture [3, 4]. In modern practice, electrical stimulation (versus manual manipulation) of acupoints, that is, electroacupuncture, is increasingly gaining popularity among various acupunctural modalities. Both animal and clinical studies indicate that acupuncture is effective in certain kinds of epilepsy. As compared to the conventional Western medicine and surgical treatments, the significant advantages of acupuncture treatment include its safety, convenience, and minimal side effects [5–7].

Acupuncture exerts its antiepileptic effect through normalization of the disrupted neuronal excitability [1]. Some acupuncture-induced effects involve the activation of the opioid system [8–13]. We have previously demonstrated that electroacupuncture has a neuroprotective role in the brain against ischemic injury via the *δ*-opioid system [14, 15]. DOR expression/activation inhibits Na^+^ channel activity [16, 17] and thus attenuates Na^+^-K^+^ homeostasis of the cortex under normoxic/hypoxic conditions [16, 18–22]. Since the electrolyte homeostasis (e.g., Na^+^ Ca^2+^, K^+^, and Cl^−^) gets disrupted between the intra- and extracellular space during epileptic seizures, and since Na^+^ channel upregulation participates in the pathological changes of several neurological disorders such as hypoxic/ischemic injury and epilepsy [23–26], acupuncture may attenuate epileptic seizures through the DOR-mediated inhibition of the Na^+^ channels [1]. Research in this new field may help us in the pursuit of novel therapeutic solutions for epileptic hyperexcitability and seizures.

## 2. Pathological Genesis of Epileptic Brain Hyperexcitability

The most prominent feature of epilepsy is the hyperexcitability in the brain. At least two major factors contribute to the hyperexcitability in the epileptic brain. The first and also the most significant contributor is the altered intrinsic electrogenic properties of the neurons (neuronal excitability), which depend on the functioning of membrane ion channels, namely Na^+^, K^+^, and Ca^2+^ channels. The second factor is the synaptic imbalance that involves disruption of chemical transmission from the neighboring cells within the network via ligand-gated ion channels and G-protein-coupled metabotropic receptors and rapid electrical transmission via gap junctions (network excitability) [2]. For both major factors, epilepsy results from the abnormal activity in the neuronal networks. However, hyperexcitability due to altered ion-channel function contributes to the seizure-prone state [25]. 

Numerous studies have shown the existence of neuronal networks of epilepsy in the epileptic patients with the aid of EEG-functional magnetic resonance imaging (EEG-fMRI) [1, 27–31]. In epilepsy, synaptic input from neighboring cells within the network is disrupted, which results in a progressive increase in excitability and epileptogenesis. This imbalanced neurotransmission between excitatory and inhibitory activities is the most prominent feature. Exaggerated glutamatergic excitatory transmission, or decreased GABAergic inhibitory transmission between the inhibitory and the excitatory systems, or a combination of these two can lead to an overexcitation of the neurons. A causal association between such imbalances in the neurotransmitter activity has been causatively linked to seizure activity and the development of chronic epilepsy [1]. In addition, aberrant fiber sprouting and robust synaptic reorganization, due to the neuronal injury/loss associated with brain damage under conditions such as status epilepticus, stroke, brain trauma, and developmental malformation or deafferentation often observed in the hippocampus and cortex, also instigate a recurrent excitatory circuit by forming synapses on the dendrites of neighboring neurons (e.g., granule cells in hippocampus). A small cluster of pathologically interconnected neurons in this aberrant recurrent network are capable of generating powerful hypersynchronous bursts of discharges, initiating epileptogenesis via a kindling effect and development of epileptic discharges that spread throughout the limbic system, and eventually resulting in temporal lobe epilepsy [1, 32–34].

Ion channels in the neuronal membrane have a critical impact on neuronal excitability. Ion channels, especially voltage-gated Na^+^, K^+^, and Ca^2+^ channels, are functional proteins embedded in the plasma membrane. They are critical determinants of neuronal excitability as they influence the generation and propagation of action potential in the neurons. Action potential is the cellular language by which neurons communicate with one another. During the firing of an action potential, the voltage-dependent Na^+^ channels dominate the explosive, regenerative activation of inward currents during the rising phase, while a fraction of the voltage-dependent K^+^ channels chiefly contribute to the repolarization and hyperpolarizing overshoot phase of the action potential. Voltage-dependent Ca^2+^ channels generally make little contribution to the rising phase of an action potential because their activation kinetics are slower than Na^+^ channels. However, Ca^2+^ entry through voltage-gated Ca^2+^ channels activates Ca^2+^-activated K^+^ channels that indirectly contribute to the late repolarization and after hyperpolarization, which follows the rising phase of an action potential [35]. Basically, voltage-gated Na^+^ channels along with K^+^ and Ca^2+^ channels determine the capacity of action potential generation, the shape of an action potential, and the firing rate and therefore have a great impact on neuronal excitability. Na^+^ channel states (opening, closing, and inactivation) make a yes-or-no decision regarding the firing of an action potential, and the channel kinetics (activation and deactivation) and current density play an essential role in the amplitude and duration of an action potential. Therefore, abnormities of the function (opening, closing, and inactivation), expression, or structure (e.g., mutation) of ion channels may be responsible for the hyperexcitability of neurons and contribute to the consequent epilepsy.

## 3. Clinical Practice of Acupuncture Therapy for Epilepsy

 Acupuncture treatment for epilepsy has been employed for thousands of years through all of the dynasties in China [50]. The first known description of epilepsy and acupuncture therapy appeared in *Huang Di Nei Jing* (The Yellow Emperor's classic of Internal Medicine), an ancient Chinese medicine book written by a group of Chinese physicians around 770–221 B.C. [50, 51]. Ancient acupuncturists maintained a memorandum of their successful cases based on clinical improvement in their clinical trials. They focused on controlling seizures and compared different acupuncture methods (acupuncture alone with acupuncture plus other therapies) to find how to better control seizures. Therefore, in a way they were using people rather than animals first to perform their experiments [50]. There is no doubt that ancient acupuncturists/physicians made important observations in validating the effects of acupuncture on epilepsy, though mostly through their personal experiences. With the aid of advanced techniques, many modern scientific researchers have demonstrated the antiepileptic effects of acupuncture in animal studies [52–60]. For example, we found that electroacupuncture stimulation of Jinsuo (GV-8) and Fengfu (GV-16) significantly improves epileptic EEG and seizure behaviors through an increase in endogenous melatonin levels in a penicillin-induced rat model [52]. Furthermore, we found in a kainic acid-induced seizure model that electroacupuncture attenuates epileptic seizures, which is relatively specific to stimulation parameters and acupoints [55, 60]. Our findings show that (1) low- or high-frequency electroacupuncture at different acupoints, for example, Renzhong (GV-26) plus Dazhui (GV-14), Jinsuo (GV-8) plus Yaoqi (EXB-9), Neiguan (PC-6) plus Quchi (LI-11), and Fenglong (ST-40) plus Yongquan (KI-1), reduced epileptic seizures with an exception of low-frequency electroacupuncture at Neiguan (PC-6) and Quchi (LI-11); (2) low-frequency electroacupuncture induced a better effect at Fenglong (ST-40) plus Yongquan (KI-1) than other acupoints; (3) there was no significant difference in effects of high-frequency electroacupuncture at these acupoints; and (4) high-frequency electroacupuncture elicited a greater effect than low-frequency electroacupuncture, with an exception of Jinsuo (GV-8) + Yaoqi (EXB-9). The electroacupuncture-induced attenuation appeared 1–1.5 hours after electroacupuncture with no appreciable effect in either EEG or behavioral tests during the first hour after electroacupuncture [55, 60]. 

Despite substantial evidence from animal research in support of antiepileptic effects of acupuncture, a large number of clinical studies have also shown that patients have benefited from acupuncture for control of their seizures, especially in cases with refractory epilepsy [36–49], though results from some studies beg to differ [61]. 

These are extremely heterogonous clinical case series [36–49]. Since many of these reports were written in Chinese and are not understood or easily available to Western peers, we have already summarized the salient information from these reports in Table 1. As shown in this table, the patients treated varied over a range of age from infants to elderly, and the type of epilepsy from absence seizure, febrile convulsion, and generalized clonic-tonic seizure, to even status epilepticus. The case numbers reported also vary from a few to over one hundred, many of which were resistant to antiepileptic drugs. The acupoints and acupuncture methods used in these reports were highly heterogeneous as well. The overall treatment effects of acupuncture are principally manifested by the reduction in symptoms (e.g., a reduction of seizure frequency, shortness of episodes, etc.), functional recovery (e.g., smoothed breath from shortened, quickened, and occasionally stopped breath, recovery from unconsciousness, etc.), improved EEG (e.g., reduction of spike wave, desynchronization, etc.), and/or decreased epilepsy scores. The outcome of acupuncture therapy in these reports is relatively significant, and the total effective rate of treatment is as high as up to 98%.

For the report that was unable to prove a beneficial effect of acupuncture in chronic intractable epilepsy [61], as the authors acknowledged, the negative results could in part be ascribed to the small sample size for this observation [61]. Also, acupoints selected and manipulation/stimulation methods adopted (e.g., frequency, intensity, duration of swift rotation, lifting, thrusting, and retaining) may also be responsible for this observation. In this respect, as has mentioned earlier in this section, electroacupunctural attenuation of epileptic seizures is relatively specific to stimulation parameters and acupoints in our animal studies [60]. 

## 4. Potential Mechanisms of Acupuncture Inhibition of Neuronal Hyperexcitability

### 4.1. Acupuncture and The Opioid System

Classic opioid systems include endogenous peptides and *δ*-, *μ*-, and *κ*-opioid receptors (DOR, MOR, and KOR, resp.). Endogenous opioid peptides enkephalin, *β*-endorphin, and dynorphin in the brain preferentially bind to DOR, MOR, and KOR, respectively, under physiological concentrations and have multiple functions in the brain. Acupuncture and electroacupuncture have been well recognized to regulate endogenous opioid systems in the central nervous system [8–13, 62].

Along with other biomediators (neurotransmitters, neuromodulators, and/or signaling molecules) [1], the opioid system is also involved in the anticonvulsant effect of acupuncture, though the role of different opioid systems in the acupuncture suppression of epilepsy appears to be very complex. 

Some studies show that the blockade of the opioid system in the brain can diminish, while its activation enhances, inhibitory effects of acupuncture on epilepsy. In this regard, Wu and his coworkers reported that in a rat model of penicillin-induced seizures, the inhibitory effect of electroacupuncture on cortical epileptiform discharges could be reversed by the injection of a broad spectrum opioid receptor antagonist, naloxone, via various routes including microinjection into the periaqueductal gray matter of the midbrain, accumbens or other nuclei, intraperitoneal, and intracerebroventricular injection, or even direct injection into the cortical site where penicillin was applied. While naloxone injection into the reticular formation of midbrain and the reticular nucleus of the thalamus had little effect on acupuncture attenuation of epilepsy, injection of naloxone into the above-mentioned sites without application of penicillin on the cortex did not induce epileptic burst [63, 64], suggesting a specific activation of the opioid system by electroacupuncture in the epileptic brain. In the hippocampus, activation of KOR with U50488H enhances, while blockade of KOR with antagonist MR2266 or antidynorphin serum abates the antiepileptic effect of electroacupuncture in electroconvulsive rats [65]. Therefore, the authors concluded that endogenously released opioid peptides mediate the suppressive effect of electroacupuncture on seizures [63, 64]. 

In support of the above viewpoint [63, 64], enhanced biosynthesis of central enkephalin by application of electroacupuncture has been found in the rat brain [66]. In the rat brain of experimental seizures model, hippocampal dynorphin concentration and dynorphin immuoreactivity in hippocampal mossy fiber and hilus are increased by electroacupuncture [67–69]. Also, the seizure-associated increase in leu-enkephalin and *β*-endorphin levels in the hippocampus and augmented expression of preproenkephalin mRNA in several brain regions (entorhinal cortex, subiculum, hippocampal CA1 area, amygdaloidal nucleus, and piriform cortex) in rats are suppressed by electroacupuncture [68, 70–72]. Therefore, acupuncture can suppress epilepsy by regulating the synthesis and release of endogenous opioids.

Acupuncture can also regulate the expression and activity of opioid receptors. Radioreceptor-binding assay combined with autoradiography revealed that repeated electroconvulsive shock resulted in epileptiform EEG, behavioral seizures, and an accompanied increase in the opioid receptor densities in several brain regions (caudate nucleus, hippocampus, habenula nucleus, and amygdale) in rats. Electroacupuncture at Fengfu (GV-16) and Jingsuo (GV-8) was found to suppress the seizure activities and significantly decrease the opioid receptor densities in these brain regions except the caudate nucleus [73]. 

Electroacupuncture can also upregulate DOR expression in the ischemic brain and thus protect the brain from ischemic injury [15] that can cause epilepsy secondary to cerebral infarction [74]. Most interestingly, direct subacute high-frequency electrical stimulation of the parahippocampal cortex in patients with intractable medial temporal lobe epilepsy also produces an antiepileptic effect, which is associated with reduced opioid peptide binding including ^3^H-DAMGO, ^3^H-DPDPE, and ^3^H-nociceptin (the ligand for MOR, DOR, and a fourth opioid, nociception receptor, resp.) in the same brain regions in patients with epilepsy [75]. 

Therefore, it is evident in both animals and patients with epilepsy that acupuncture can regulate the activities of endogenous opioids and their receptors in the brain, thereby exerting its antiepileptic effects.

### 4.2. Opioid Receptors and Na^+^ Channels

Opioid receptors are members of the G-protein-coupled receptor superfamily [76], and can functionally couple with ion channels, including Na^+^ channels [16, 17, 77]. Our previous data implied an interaction between opioid receptors and Na^+^ channels. For example, we observed that DOR downregulation [78] is associated with an upregulation of voltage-gated Na^+^ channels in a mutant brain with epileptic seizures [24]. An increased Na^+^channel density [23] along with decreased DOR density [79] occurred in hypoxia-exposed brain. Activation of presynaptic DOR by enkephalin prevents the increase in neuronal Nav1.7 in the dorsal root ganglia, which relieves pain in diabetic neuropathy [17]. More recently, our studies indicated that DOR activation attenuates hypoxic K^+^-Na^+^ homeostasis, which largely relies on DOR inhibition of Na^+^ influx through Na^+^ channels [16, 18–22]. These results suggest that under pathological conditions DOR could mediate an inhibitory regulation of Na^+^ channels in the brain. 

Furthermore, our direct evidence gained from electrophysiological studies shows that activation of DOR indeed inhibits Na^+^ channel activity [16]. Remy et al. observed that SNC80 (1–1000 *μ*M), a putative DOR agonist, reduced the maximal Na^+^ current amplitude in a dose-dependent manner and selectively prolonged the course of recovery from slow inactivation without effects on fast inactivation processes [80]. However, the authors concluded that this effect was opioid receptor independent since the effects of SNC80 were not mimicked by another DOR agonist DPDPE (10 *μ*M) and were not inhibited by high concentrations of opioid receptor antagonists, naloxone (50–300 *μ*M), and naltrindole (10 and 100 *μ*M) [80]. This conclusion is arguable due to several important issues (e.g., experimental procedures, specificity and dose of drugs used, etc.) (see review [81]). We recently took the advantages of *Xenopus* oocytes with coexpressed DOR and Na^+^ channels to explore a “pure” interaction between DOR and Na^+^ channels, and found the following: (1) Nav1.2 expression induced tetrodotoxin- (TTX-) sensitive inward currents; (2) DOR expression reduced the inward currents; (3) activation of DOR reduced the amplitude of the current and rightward shifted the activation curve of the current in the oocytes with both Nav1.2 and DOR, but not in oocytes with Nav1.2 alone; (4) the DOR agonist-induced inhibition of Nav1.2 currents was in a dose-dependent manner and saturable; and (5) the selective DOR agonist had no effect on naive oocytes. These findings present the first demonstration that activation of DOR inhibits Na^+^ channel function by decreasing the amplitude of Na^+^ currents and increasing its threshold for activation [16]. Similar inhibitory effect of Na^+^ channels is also found on MOR and KOR. For example, in acutely isolated cortical neurons, the application of 1 *μ*M of DAMGO, a specific MOR agonist, caused a decrease in the Na^+^ current amplitude to approximately 79% of the controls. Moreover, DAMGO decreased the maximum current activation rate, prolonged its time-dependent inactivation, shifted the half inactivation voltage from −63.4 mV to −71.5 mV, and prolonged the time constant of recovery from inactivation from 5.4 ms to 7.4 ms [77]. DAMGO also inhibited TTX-resistant voltage-dependent Na^+^ current in dorsal root ganglion neurons [82]. U50488, a KOR agonist, decreases voltage-activated Na^+^ currents in colon sensory neurons [83]. Therefore, it seems that inhibition of Na^+^ channel activities, which depends on signal molecules such as protein kinases [17, 22, 77], is one of the common characteristics of opioid receptors.

### 4.3. Na^+^ Channels and Epileptic Hyperexcitability

As shown by previous reviews [84, 85], brain Na^+^ channels consist of a 260 kDa *α* subunit with two auxiliary *β* subunits. The *α* subunit forms the ion-conducting pore and the activation and inactivation gates that regulate voltage-dependent sodium flux across the plasma membrane, while the *β* subunits modify the kinetics and voltage dependence of the gates. Until now at least nine *α* subtypes (Nav1.1–Nav1.9) and four *β* subtypes (*β*1–*β*4) have been found to express in the excitable cells (Table 2). Nav1.1, Nav1.2, Nav1.3, and Nav1.6 are the primary subtypes in the central nervous system [26, 84–87]. The developmental expression and cellular localization of these subtypes are different in the brain. Nav1.1 expression is first detectable at postnatal day 7, increases during the third postnatal week, and peaks at the end of the first postnatal month, after which levels decrease by about 50% in the adult. Nav1.2 expression also increases during the third postnatal week and continues to increase thereafter, until the maximal level is reached in adulthood. In rodents, Nav1.3 channels are highly expressed in the brain during the embryonic period, peak at birth, and decline after birth as Nav1.1 and Nav1.2 channels take over, but remain detectable at a lower level during adulthood. Nav1.3 remains at comparatively higher levels in the human adult brain in adulthood. Nav1.1 and Nav1.3 are primary localized in the cell body and are preferentially expressed in the GABAergic neurons. Nav1.2 is particularly highly expressed in the unmyelinated or premyelinated axons and dendrites. Nav1.6 subtype is expressed at the nodes of Ranvier and the initial segments of axons, as well as in the somata and dendrites of many projection neurons [26, 84–87]. Sodium channels are responsible for the initiation and propagation of action potential and influence the subthreshold electrophysiology. Therefore, they are crucial determinants of intrinsic neuronal excitability [25, 35, 86]. Since epilepsy is regarded as an “electrical storm” of brain hyperexcitability [86], altered density or biophysical properties of Na^+^ channels may have important consequences on the neuronal excitability and may contribute to the pathophysiology of brain diseases associated with altered excitability, such as epilepsy [26, 86, 88–90]. The importance of Na^+^ channels in brain hyperexcitability and the consequent epilepsy is further supported by the fact that a large number of antiepileptic drugs exert their antiepileptic effect by interacting with Na^+^ channels [2, 91].

#### 4.3.1. Expressional and Functional Alterations in Na^+^ Channels during Epilepsy

Accumulating evidence indicates that altered expression and functional regulation of Na^+^ channels in the neurons play an important role in the brain hyperexcitability and epileptic phenotype in both acquired and inherited epilepsy. In the cortex of a genetically seizure susceptible E1 mouse brain and spontaneously epileptic rat hippocampus, a significant increase in total Na^+^ channel mRNA and protein, as well as in Nav1.1, Nav1.3, and *β*1 subunits, was observed to contribute to the generation of epileptiform activity and the observed seizure phenotypes [92, 93]. The expression of Nav1.1 in hippocampal CA1 and that of Nav1.2 in the cortical neurons were found to be significantly increased, which was accompanied with increased neuronal excitability and spontaneous epileptic seizures in the Na^+^/H^+^ exchanger null mutant mouse [24, 94]. In a kindling seizure model, selectively increased expression of Nav1.6 mRNA and protein in hippocampal CA3 neurons and Nav1.6 immunoreactivity in the medial entorhinal neurons resulted in hyperexcitability in these brain regions [95, 96]. In acute status epilepticus models, a transient upregulation of Na^+^ channel mRNAs encoding Nav1.2 and Nav1.3 subunits was observed in the hippocampal neurons [97, 98]. In contrast, the rats with status epilepticus and chronically developed spontaneous epileptic seizures showed a selectively persistent down-regulation of Nav1.2, Nav1.6, and *β*1 subunits, as well as short-term down-regulation of *β*2 subunit. In addition, an increased excitability, manifested by the augmented window current due to the significant positive shift of inactivation potential and negative shift of activation potential and the resultant increased overlap between the activation and inactivation curve, was observed in the neurons of the hippocampal dentate gyrus [99]. This phenomenon may be caused by the down-regulation of *β* subunit expression, since *β*1/*β*2 subunit, if co-expressed with *α* subunits, favors inactivation, accelerates recovery of Na^+^ currents [100–102], which increases the number of Na^+^ channels available to be activated, and thus increases the firing rate. In human brain with temporal lobe epilepsy, Nav1.3 mRNA in the pyramidal cells of hippocampal CA4 area is significantly upregulated, and Nav1.2 mRNA in the remaining pyramidal cells of hippocampal CA1, CA2, and CA3 areas is largely downregulated, while Nav1.1 and Nav1.6 do not show any differences in their expression in the hippocampus [103]. *SCN7A* gene that encodes atypical Na^+^ channels (Na_X_) was recently reported to be increasingly and persistently expressed in the pyramidal neurons and astrocytes of the hippocampal CA1 and CA3 areas in patients with drug-resistant temporal lobe epilepsy and epileptic rats and is possibly responsible for the enhanced brain excitability and epileptogenesis [104]. In summary, the increased amplitude and density of the voltage-dependent Na^+^ currents, shortened phase of inactivation, and enhanced window currents due to a shift towards depolarization of inactivation currents and more negative activation has been associated with neuronal hyperexcitability and the development of some types of epilepsy [24, 94, 99, 105, 106]. Na^+^ channels, even in the few that fail to inactivate, carry the persistent fraction of Na^+^ currents (that are not sensitive to TTX), though small, and may drive the membrane towards the firing threshold. Especially under physiological conditions, the persistent sodium currents serve to amplify or spatially integrate synaptic potentials, allow excitable cells to generate subthreshold oscillations, reduce the threshold for repetitive action potential firing, and therefore increase excitability of neurons associated with epilepsy [25, 95, 96, 107, 108]. Despite the differences in epilepsy types/models and Na^+^channel subtypes in these reports, these findings suggest altered expression and functional regulation of Na^+^ channels (that carry Na^+^ currents including both TTX-sensitive and -resistant ones) are critically involved in brain hyperexcitability and the pathology of epilepsy. 

#### 4.3.2. Insights from Genetic Epilepsy Model

Genetic factors are important for the intrinsic excitability of neurons. Studies on Na^+^ channel mutation in the genetic epilepsy model broaden the view on the roles and the underlying mechanisms of Na^+^ channels in brain hyperexcitability and the pathophysiology of epilepsy. Gene mutations that result in channel dysfunction (channelopathies) play an essential role in neuronal excitability, leading to the development of a variety of epilepsy syndromes. The most convincing data on the role of Na^+^ channels in brain hyperexcitability and epileptogenesis comes from the identification of several hundred mutations of Na^+^channels which lead to inherited epileptic syndromes ranging in severity from relatively mild disorders such as benign familial neonatal-infantile seizures (BFNIS), simple febrile seizures, and generalized epilepsy with febrile seizure plus (GEFS+), to severe epileptic encephalopathy such as severe myoclonic epilepsy of infancy (SMEI, also called Dravet's Syndrome), SMEI borderline, and intractable childhood epilepsy with generalized tonic-clonic seizures (see reviews [25, 86–89]).

So far, several hundred mutations of many Na^+^ channel subtypes, including Nav1.1 (*SCN1A*), Nav1.2 (*SCN2A*), Nav1.3 (*SCN3*A), Nav1.6 (*SCN8A*), even Nav1.7 (*SCN9A*, which is predominantly expressed in peripheral nervous system), and *β*1 subunit (*SCN1B*), have been causally linked to a variety of genetic epilepsies [89]. However, the genotype-phenotype correlations for Na^+^ channel epilepsy are very complicated with high heterogeneity. This heterogeneity of genotype-phenotype correlation for Na^+^ channel epilepsy is reflected in the observation that the same gene mutation may result in different phenotypes of epilepsy, and in turn, a single phenotype may be a result of different gene mutations of Na^+^ channels. For example, mutations in *SCN1A* have been reported to cause epilepsy with the symptoms ranging from febrile seizures and GEFS+ to SMEI, and mutations in *SCN2A* are identified to cause BFNIS, GEFS+, SMEI, and intractable epilepsy with mental decline [86–89]. On the other hand, Dravet's Syndrome is reported to have mutations in *SCN1A*, *SCN2A,* and *SCN1B*, and the mutations in both *SCN1A* and *SCN2A* lead to GEFS+ [87, 89]. Among all the mutations, Nav1.1 mutations account for the majority of established epilepsy syndromes in children [86–89, 109]. Approximately 30 *SCN1A* mutations have been identified to account for GEFS+ and all of them are missense mutations. Many other missense mutations in *SCN1A* also contribute to Dravet's Syndrome, but most of the complications of Dravet's Syndrome result from *SCN1A* mutations caused by frame shift, nonsense, and splice-site mutations, which lead to a truncated protein and haploinsufficiency of *SCN1A* [86–90, 109, 110]. Unlike *SCN1A*-GEFS+ mutations that show a Mendelian inheritance pattern within affected families, most *SCN1A*-SMEI mutations occur *de novo* in the affected child.

Na^+^ channel mutations may result in either gain of function or loss of function that has been proposed to, respectively, increase and decrease the neuronal excitability [87]. The gain of function of Na^+^ channels is manifested by the increased current density, negative shift of activation, positive shift of inactivation, enhanced persistent Na^+^ currents, or mixed effects on channel kinetics but with the net effect of an increase in activity. The loss of function of Na^+^ channels is reflected by the changes that are opposite to those seen with gain of function. However, both gain of function and loss of function in Nav1.1 can predispose the brain to abnormal excitability, that is, brain hyperexcitability and consequent epilepsy syndrome. The increase in neuronal excitability due to gain of function in Na^+^ channels is quite straightforward to be understood and is further supported by the fact that many antiepileptic drugs developed are Na^+^ channel blockers [2, 91]. It is very surprising, however, that “loss of function” in Na^+^ channels also makes the brain very hyperexcitable although it decreases the neuronal excitability. Fortunately, this mystery was unveiled recently with the targeted knock-out mice and rats carrying mutated *SCN1A* which developed epileptic seizures (SMEI and GEFS+) and sporadically died within the first postnatal month for the SMEI mice [90, 111–114]. Immunohistochemical analysis revealed that in a developing rodent brain, Nav1.1 was predominantly expressed in the inhibitory GABAergic interneurons of the neocortex and hippocampus, as well as the cerebellar GABAergic Purkinje cells that serve as the output pathway for information on movement, coordination, and balance from the cerebellar cortex [111–114]. Mutations or deletion of Nav1.1 lead to the loss of sustained high-frequency firing of action potential and excitability in the hippocampal and cortical inhibitory interneurons and Purkinje neurons, which allows hyperexcitability of principal neurons (e.g., dentate granule cells and pyramidal neurons) in the neuronal networks, thus leading to brain hyperexcitability and subsequently epilepsy. A unified loss of function hypothesis for Nav1.1 genetic epilepsies has been proposed [86, 88]. According to this hypothesis, the severity of epileptic phenotypes of Nav1.1 mutations is dependent on the extent of Nav1.1 functional damage due to mutations, as, for instance, the spectrum of severity of Nav1.1-associated forms of epilepsy results from an increasing severity of loss of function mutations in Nav1.1 channels and increasing impairment of action potential firing of the GABAergic inhibitory neurons [88]. As mentioned earlier, mild impairment of Nav1.1 channel function causes febrile seizures and moderate to severe impairment of Nav1.1 function due to missense or nonsense mutations causing GEFS+ and SMEI [86, 88].

#### 4.3.3. Na^+^ Channel Based Neuronal and Network Excitability

The above discussion on altered expression and functions as well as mutations in Na^+^ channels strongly supports the belief that abnormal Na^+^ channel activities are critically involved in neuronal hyperexcitability and the pathology of epilepsy. However, it should be pointed out that emphasizing the important roles of Na^+^ channels in the intrinsic neuronal excitability and the consequent epilepsy does not mean that Na^+^ channels hold any less importance in the network excitability. As we elaborated earlier, both neuronal excitability and network excitability contribute to the hyperexcitability in the epileptic brain. Since epileptic seizures are regarded as an “electrical storm” of brain hyperexcitability [86], they involve not only intrinsic neuronal properties, but also a cluster of anatomically and functionally associated neurons that form epileptic networks and propagate the extremely synchronized “electrical storm” within the networks [1]. Therefore, it is important to realize that Na^+^ channels regulate the brain excitability by altering both intrinsic neuronal and network excitability. The genetic models of SMEI show that the Nav1.1 mutation or deletion affects the inhibitory interneurons in the hippocampus and cortex [90, 111, 113]. Even though the excitatory transmission is not affected, the balance between inhibition and excitation within the networks is severely disrupted [111, 113]. In this model, though the embryonic Nav1.3 channels were found to be upregulated probably as a partial compensatory response to the impaired inhibition and disrupted balance between inhibitory and excitatory transmission [111], the network excitability is greatly enhanced due to Nav1.1 mutation that leads to a loss of sustained high-frequency firing of action potential in the hippocampal and cortical inhibitory interneurons and a limited compensatory capacity of Nav1.3 channels, thereby, making the brain very hyperexcitable. Therefore, Na^+^ channels play a central role in epileptogenesis, which involves interplay of both intrinsic neuronal properties and network activities. 

## 5. Concluding Remarks

Na^+^ channel expressional and functional upregulation has been demonstrated to be critical to epileptic hyperexcitability and seizures, while the inhibitory regulation of Na^+^ channels by DOR [16, 20–22, 81] may contribute to the proper control of neuronal excitability. An impairment of such a balancing mechanism, for example, Na^+^ channel upregulation and/or DOR downregulation in genetic or acquired conditions, may lead to neuronal dysfunction and eventually neurological diseases, especially epileptic seizures. Indeed, Na^+^ channel dysregulation has been casually linked to human epilepsy and well demonstrated in epileptic animals with abundant supporting evidence. For example, in the mutant brain exhibiting spontaneous epilepsy, Na^+^ channel was upregulated [24], while DOR was downregulated in the same brain [78], suggesting a potential role of DOR impairment in the pathophysiology of epilepsy associated with genetic abnormality. 

**Figure 1:**
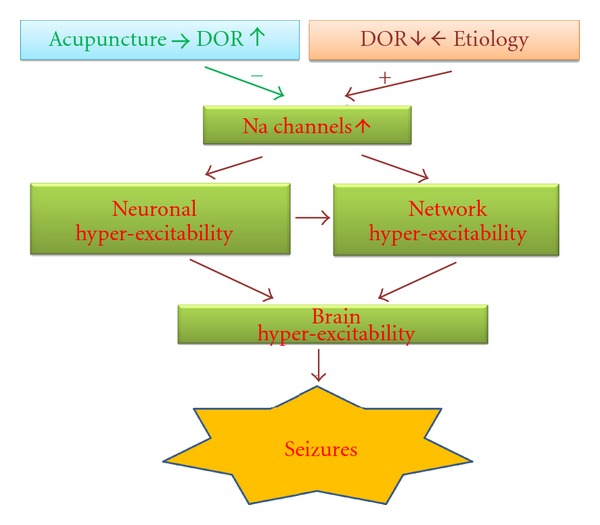
Schematic demonstration of the potential relation between acupuncture, opioid, and Na^+^ channels in the regulation of brain hyperexcitability and epileptic seizures. Acupuncture can regulate the levels of endogenous opioids and their receptors in the brain. The released opioids activate *δ*-opioid receptors, and Na^+^ channels are inhibited by activated *δ*-opioid receptors via signaling molecules such as PKC. Thus the neuronal discharges are inhibited and overexcited brain is “cooled” leading to the termination of epilepsy.

Since Na^+^ channel upregulation contributes greatly to some kinds of epileptic hyperexcitability that leads to epilepsies, the DOR-mediated inhibition of Na^+^ channels could provide a novel clue to open a vast potential of solutions to epileptic seizures. In fact, many antiepileptic drugs are actually inhibitors of Na^+^ channels [2, 91]. Moreover, acupuncture can regulate the activities of endogenous opioids and their receptors in both animals and patients with epilepsy, thus exerting its antiepileptic effects. Therefore, stimulating appropriate acupoints with suitable manipulations may be a useful strategy for the treatment of epilepsy.  Figure 1 presents a schematic demonstration regarding the interaction between acupuncture, opioids and Na^+^ channels in regulation of hyperexcitability and epileptic seizures in the brain. 

However, some issues need attention with regard to the association between acupuncture therapy for epilepsy and the role of opioids, and Na^+^ channels. As previously discussed, loss of function of Na^+^ channels in the inhibitory interneurons can cause brain hyperexcitability and epilepsy. Therefore, it is possible that activation of the opioid system by acupuncture causes inhibition of Na^+^ channel activity in inhibitory interneurons, which may further aggravate the symptoms of epilepsy. Despite the demonstration of an antiepileptic effect following DOR activation in some studies [115–118], several other reports showed opposite results. For example, SNC80, a putative DOR agonist, is proconvulsive [119, 120] though it is reported to inhibit Na^+^ channel activity [80]. The reasons for the complex and mixed effects of the *δ*-opioid system on seizures are not well clarified yet, but could be partially related to multiple factors like animal species (e.g., proconvulsive in rats but has little effect in rhesus monkeys) [119–121], seizure types, the methods of drug administration [122], dose used [123], target neurons, and so forth (also see [1]). Among these factors, the location of DOR on the target neuron seems critical and important. In the hippocampus, both granule cells and inhibitory GABAergic interneurons express DOR [124, 125]. DOR activation in the granule cells inhibits voltage-gated Na^+^ channels and thus lowers the excitability of granule neurons [80], which reduces excitatory transmission in the epileptic network and subsequently suppresses seizures. However, DOR activation in inhibitory interneurons leading to the inhibition of Na^+^ channels, as observed by Remy et al. [80] in granule neurons, may result in facilitation, rather than suppression, of seizures via postsynaptic deinhibition [126, 127], as has been observed [119, 120]. Therefore, selective activation/inhibition of the opioid system in certain locations of the epileptic networks with acupuncture therapy is critical but also challengeable for the therapeutic outcome. It has been shown that in seizure rat models, acupuncture increases dynorphin synthesis and release, enhances the activity of KOR (which is abundantly distributed in hippocampal granule cell and perforant path) [126, 127] in the hippocampus, and decreases the DOR activity, synthesis, and release of enkephalin (which mainly influences inhibitory interneuron activity) [124, 125]. Therefore, acupuncture balances the inhibition and excitation in the network and thus suppresses the seizures [1]. In this way, acupuncture exerts its antiepileptic effects by normalizing the disrupted neuronal and network excitability (by lowering the overexcited neuronal activity through multiple strategies like enhancing the inhibitory system and attenuating the excitatory system in the brain via regulation of the activities of opioid-Na^+^ channels), since the effects of acupuncture on disrupted neuronal function are bidirectional depending on the alterations in the brain functions and activities [128]. We believe that a further clarification on the correlations between acupuncture, opioids, and Na^+^ channels can better help us explore the mystery of acupuncture therapy on epilepsy. 

## Figures and Tables

**Table 1:** Clinical reports on acupuncture therapy for epilepsy from some Chinese literature.

Ref.	Patients	Age	Types of epilepsy	Acupuncture methods and Acupoints	Therapeutic assessment	Outcome
[36]	114 cases and 8 healthy control	Mean 19 yrs. (6–68) with a history of epilepsy for 1 mo.–35 yr.	Various (Grand mal, petit mal, focal, abdominal pain induced, psychomotor induced, mixed)	Scalp acupuncture (thoracic region, motor region, chorea and parkinsonism control region, foot motor sensory region, optic region) Body acupuncture (HT-7, LR-3, GV-26, GV-20, GB-20, LI-4, ST-36)	EEG; bell sound and verbal suggestion; response to pinching of the neck skin	72.6% with EEG changes mainly as asynchronism (reduction or cessation of epileptic discharges)

[37]	98 cases	Mean 27 yrs. (2–52) with a history of epilepsy for Ave. 17 yr.	Not specified (epileptic attack or EEG confirmed epilepsy)	Scalp acupuncture (Motor area, psychic area, sensory area) Once daily for 15 days as a session, 2-3 sessions in total, 7-day break between sessions, needle retention 30 min	*Markedly effective* (>75% seizure frequency reduction, or seizure controlled) *Effective * ** **(50–75% reduction, seizure less severe and interval prolonged) *Slightly effective * ** **(25–50% reduction) *No effect * ** **(<25% reduction)	66.3% markedly effective; 23.5% effective; 5.1% effective; 5.1% no effect The overall effective rate is 89.8%

[38]	8 cases	5–16 yrs with a history of epilepsy for 1 mo.–7 yr.	Status epilepticus	Manual acupuncture (LI-4, LR-3, Gv-26, GV-20, KI-1, EX-UE-11, PC-5, HT-7, RN-4, ST-40, EX-HN-3, GB-20, SP-6)	*Symptoms * ** **(unconscious, white form in mouth, cyanotic face, spastic and convulsive in limbs, short, quick breath with occasional stops, sputum in throat, uncontrolled urine)	Symptoms controlled with 10 min of acupuncture without relapse in 2–8 yr. followup

[39]	78 cases	Mean 24.7 yr. (17–39 yr.)	narcotic abstinence-induced seizures	Manual acupuncture Acupoint: PC-6 Once daily for 10 days, needle retention 30 min with 2 times of stimulation	*Markedly effective* (the symptoms of drug addiction and abstaining-induced seizures disappear, and no relapse in 6 mo.) *Effective* (alleviated symptoms, occasional relapse in 1 mo.) *No effect* (no relief of symptoms, tranquilizer needed for control of symptoms)	70.51% markedly effective; 23.08% effective; 6.41% no effect The overall effective rate is 93.59%

[40]	129 cases (64-catgut implantation group, 65-AED controls)	Mean 21.8 ± 12.0 yrs with a history of epilepsy for Ave. 7.4 yr.	General tonic-clonic epilepsy	Combined catgut implantation and small dose AED (GV-20, BL-18, ST-40, EX-B-9, CV-15, GB-34, BL-15) One time of implantation in every 25–30 days as a session for 4-5 sessions in total	*Controlled* (>92% of therapeutic efficacy percentile, no relapse), *Markedly effective* (70–92% of therapeutic efficacy percentile, 75% seizure frequency reduction) *Effective* (40–70% of therapeutic efficacy percentile, 50% seizure frequency reduction), *Slightly effective* (20–40% of therapeutic efficacy percentile, 25–50% seizure frequency reduction) *no effect* (<20% of therapeutic efficacy percentile, <25% seizure frequency reduction)	28.12% (versus 16.92% for control) controlled; 43.75% (versus 33.85%) markedly effective; 21.88% 9 (versus 35.38%) effective; 4.69% (versus 10.77%) slightly effective; 1.56% (versus 3.08%) not effective The overall effective rate is 93.75% (versus 86.15% for control)

[41]	290 cases (160-acupoint catgut embedding group, and 130-acupuncture group)	1–48 Yrs with a history of epilepsy for 10 d–21 yr.	Mixed epilepsy	Acupoint catgut embedding, acupuncture Primary acupoints: For catgut embedding-BL-14 penetrating to BL-15, BL-18 to BL-19, BL-20 to BL-21, EX-B-9 For acupuncture: CV-15, GV-20, EX-B-9, PC-5, ST-40 Secondary acupoints (same for two treatments): BL-12 + GV-20; or ST-36 +ST-34; or ST-40 + ST-36; or BL-17 + SP-10; or BL-23 + GV-4 One time of implantation in every 20 days as a session for 6 sessions in total For acupuncture, 1 time every other day for 6 mo., needle retention 20 min with stimulation 1 time per 5–10 min	*Markedly effective* (>75% seizure frequency reduction or no relapse in 1 yr.) *Effective* (50–75% seizure frequency reduction), improved (25–50% seizure frequency reduction), *No effect* (<25% seizure frequency reduction)	The total effective rate is 89.4% and 77.7% for catgut embedding and acupuncture group, respectively

[42]	120 cases	1.5–55 yrs with a history of epilepsy for 2 mo.–36 yr.	Various (Grand mal, petit mal, focal, abdominal pain induced, psychomotor induced, traumatic, mixed)	Primary acupoints: GV-20, DU-11, EX-B-9 Secondary acupoints: 1: GV-26 + GV-20, PC-6, LI-4, LR-3; 2: ST-36, BL-15, BL-18, BL-20, BL-23; + ST-40 or BL-62 or KI-6. Once daily for 10 days as a session, 3 sessions in total, 3–5-day break between sessions, needle retention 30 min with stimulation 1 time per 10 min	Same as Shi et al., 1987 [37]	71.7% markedly effective; 23.3% effective; 3.3% effective; 1.7% no effect The overall effective rate is 98.3%

[43]	60 cases (30-acupuncture + Xi Feng capsule group, and 30-Xi Feng capsul controls)	<5 yr–16 yrs with a history of epilepsy for < 1 yr–15 yr.	Tonic-clonic epilepsy	Combined acupuncture with Xi Feng capsule Acupoints include GV-26, GV-20, GB-20, PC-6, LR-3, ST-36 Once daily for 8 days as a session, 2 sessions in total, 2-day break between sessions, needle retention 30 min with stimulation 1 time per 10 min	*Markedly effective* (>75% reduction of seizure duration, >4 reduction of epileptic EEG score) *Effective* (50–75% reduction of seizure duration, 2–4 reduction of epileptic EEG score) *No effect* (<50% reduction of seizure duration, <2 reduction of epileptic EEG score)	96.7% (versus 90% for control) overall effective rate in seizure frequency reduction, 80% (versus 60%) in reduction of seizure duration, and 92.3% (versus 88.5%) in EEG improvement

[44]	60 cases (30-acupuncture group, and 30-AED controls)	Mean 65 yrs (40–70 yrs)	Epilepsy secondary to cerebral infarction (focal and general tonic-clonic)	Combined acupuncture and Chinese herb Acupoints include three acupoints on back created by the author (Shaofeng Guo) 1 time daily for 14 days as a session, 2 sessions in total	Controlled (no relapse) *Markedly effective* (>75% seizure frequency reduction) *Effective* (50–75% seizure frequency reduction) *No effect* (<50% seizure frequency reduction, or increased)	The overall effective rate is 93.3% (versus 80% for control) No adverse responses (mild dizziness, hypomnesia, limb numbness, weight loss and lassitude that showed in AED treatment control) appear

[45]	90 cases (30-acupuncture group, 30-catgut implantation group, 30-AED controls)	Mean age 35.02, 33.56 and 31.79 yrs, in acupuncture, catgut, and control groups, respectively, with a history of epilepsy for Ave. 7.96, 7.30, and 7.68 yr for acupuncture, catgut implantation, and control, respectively	General tonic-clonic epilepsy	Acupuncture and catgut implantation Primary acupoints include 1: GV-20 + GV-8 + ST-40; 2: BL-15 + BL-18 + GB-34; 3: BL-15 + BL-19 + LI-14 Secondary acupoints include BL-19, GB-20, BL-17, BL-21, BL-23 For catgut embedding, 1 time of embedding in every 15 days for 3 mo For acupuncture, 1 time every other day for 3 mo., needle retention 30 min with stimulation 1 time per 5–10 min	Same as Deng et al., 2001 [40]	The overall effective rate is 93.33%, 86.67%, and 76.67% for catgut implantation, acupuncture group, and control, respectively

[46]	100 cases (50-catgut implantation group, 50-AED controls)	Mean age 30.25 (versus 33.20 in controls) with a history of epilepsy for Ave. 7.71 (versus 7.33 for control) yr.	General paroxysmal epilepsy	Catgut implantation Acupoints and treatment as same as Zhang et al., 2006 [45]	Same as Deng et al., 2001 [40], Zhang et al., 2006 [45]	The overall effective rate is 94.0% and 82.0% for catgut implantation group and control, respectively

[47]	98 cases	12–63 yrs with a history of epilepsy for 5 mo.–20 yr.	Jacksonian epilepsy	Penetrating needling together with scalp acupuncture and strong/electric needling on body points GV-14 penetrating to GV-10, GV-9 to GV-8, GV-6 to GV-4, EX-B9 to GV-1, GV-24 to GV-22, GV-20 to GV-19, CV-15 to CV-12 Bilateral PC-6, ST-40, LR-3, and MS-6 Intermittent dense-loose waves with 2-3 Hz for 30–45 min every other day for 10 times as a session with a total 2 sessions and a break of 3–5 days between 2 sessions	Same as Shi et al., 1987 [37]	The total effective rate is 85.7%

[48]	80 cases (40-catgut implantation group and 40-herbal medicine controls)	6–52 yrs with a history of epilepsy for 1–15 yr.	Grand mal, petit mal, and mixed	Combined catgut implantation and herbal medicine Acupoints include GV-20, EX-B9, PC-6, CV-15 + ST-40 for phlegm, or + CV-12 for abdominal pain, or + BL-23 for uncontrolled urine 1 time of catgut implantation in every 20–30 days for 6 times	Same as Mao and Guo, 2005 [44]	The overall effective rate is 97.5% and 85.0% for catgut implantation group and control, respectively

[49]	70 cases (36 combined acupuncture and AED group, 34 AED only controls)	6 mo.–6 yr with a history of epilepsy for 1 day	Infantile febrile convulsion	Combined acupuncture and AED Acupoints include GV-26, + KI-1, LI-11, LI-4, and LU-11 for cessation of spasm, or + PC-6 and ST-36 for cessation of vomit	*Rapidly effective*: spasms cease within 1-2 min of treatment *Basically effective*: spasms cease in 3-4 min of treatment *Ineffective*: spasms cease in >5 min of treatment *Relapse*: >2 times of spasms during 1–3 days of treatment *Non relapse*: no relapse during 1–3 days of treatment	77.7% (versus 23.5% for control) rapidly effective; 8.3% (versus 55.9%) basically effective; 13.9% (versus 20.6%) ineffective The overall effective rate is 86.1% (versus 79.4% for control) Relapse rate is 8.3% (versus 32.4% for control)

Note: since many of these reports were written in Chinese and are not easily available and/or understood by Western peers, we extracted relevant information from these reports and summarized it in this table.

**Table 2:** Voltage-gated sodium channels.

Subunit	α	β
Subtypes	Nav1.1–Nav1.9	β1–β4

Location	Prevalent in the CNS:	Two β subunits associated with an *α* subunit
	Nav1.1, Nav1.2, Nav1.3, and Nav1.6	
	Abundant in muscle:	
	Nav1.4, Nav1.5	
	Primarily in peripheral nervous system:	
	Nav1.7, Nav1.8, and Nav1.9	

Cellular distribution	Primary localized in cell body:	Expressed in a complementary fashion (either β1 or β3, and β2 or β4) with α subunit
	Nav1.1 and Nav1.3	
	High expression in unmyelinated or pre myelinated axons and dendrites:	
	Nav1.2	
	Nodes of Ranvier and axon initial segments as well as in the somata and dendrites of many projection neurons:	
	Nav1.6	

Function	Forms the ion-conducting pore and activation and inactivation gates	Modify the kinetics and voltage dependence of gating Serve as cell adhesion molecules for integrating the channels into the appropriate subcellular domains

## Abbreviations

BFNIS: Benign familial neonatal-infantile seizures
DOR: *δ*-opioid receptor
GEFS+: Generalized epilepsy with febrile seizure plus
KOR: *κ*-opioid receptor
MOR: *μ*-opioid receptor
SMEI: Severe myoclonic epilepsy of infancy
TTX: Tetrodotoxin.
